# Prospective, Multicenter Evaluation of a Polyherbal Supplement alongside Standard-of-Care Treatment for Mild Knee Osteoarthritis

**DOI:** 10.1155/2021/5589597

**Published:** 2021-05-07

**Authors:** Zbigniew Żęgota, Joanna Goździk, Joanna Głogowska-Szeląg

**Affiliations:** ^1^Zbigniew Żęgota Specjalistyczny Ośrodek Leczniczo-Badawczy, Ostróda, Poland; ^2^Silmedic Ltd., Katowice, Poland; ^3^Department of Pathophysiology and Endocrinology, Medical University of Silesia, Zabrze, Poland

## Abstract

**Background:**

This study aimed to provide clinical information on general and joint performance from individuals taking Tregocel® (containing curcuminoid and extracts of the herbs *Harpagophytum procumbens*, *Boswellia serrata, Apium graveolens*, and *Zingiber officinale*) alongside a standard therapy of symptomatic mild knee osteoarthritis (OA).

**Methods:**

This was a multicenter, open-label, prospective, single-arm study, in which Tregocel® was supplemented for 36 weeks. Participants with symptomatic mild knee OA requiring pharmacologic treatment for pain were enrolled. Physical performance (6-minute walk test, WOMAC-pain and functional domain, and heel-thigh distance flexion test), general performance (WOMAC questionnaire), and VAS (Visual Analogue Scale) assessment of knee pain, as well as anti-inflammatory and analgesic medication consumption, were assessed.

**Results:**

Between January and April 2019, 107 participants were enrolled and analysed in per protocol population. Mean age was 59.7 (SD 10.8) years, and there were 68.2% women. Mean observation time was 291.1 (SD 7.7) days. Mean increase in 6MWT result observed at the end of the study was 26.0 (SD 30.4) m (*p* < 0.001). Median VAS score decreased from 60.0 (IQR 50–72) mm at the beginning of the study to 21.0 (IQR 14–30) mm after 36 weeks of product administration (*p* < 0.001). Regular knee OA medications were taken in 99.1% of subjects at baseline decreasing to 55.1% at the end of the Tregocel® supplementation.

**Conclusions:**

During Tregocel® supplementation, participants observed improved functional capacity confirmed in the distance in 6MWT and in the heel-thigh distance flexion test, decreased level of pain, and improved WOMAC scores for all domains.

## 1. Introduction

Osteoarthritis (OA) is the predominant form of arthritis and often hands, knees, feet, and hips are involved. Pain is the main symptom that leads people to present to healthcare providers with a subsequent diagnosis of OA. Data modelling from the 2017 Global Burden of Disease study estimated that there were about 303.1 million (95% CI 273.3 to 338.6 million) prevalent cases of hip and knee OA, with a 9.3% increase from 1990 to 2017 [1].

The lifetime risk of developing knee OA is estimated at about 46% [2], with an incidence that increases with age and affects more women than men. Symptomatic knee OA rates increase rapidly around 50 years of age and then level off after the age of 70 [3].

The rapid increase in OA prevalence results in a growing impact and major challenges for healthcare systems. This is very true when we realize that OA is frequently associated with disturbed sleep, depression, increased sedentary behaviour, less physical activity, obesity, and polypharmacy, leading to decreased quality of life and overall physical and mental performance. Currently, treatment of OA varies widely from lifestyle changes (e.g., weight reduction with exercise and diet), physical aids (e.g., canes or braces), physical therapies, and medications including acetaminophen, nonsteroidal anti-inflammatory drugs (NSAIDs), corticosteroids, and/or complementary and alternative medicines (CAMs) [4–7].

A proprietary form of CAM developed in Australia, called Tregocel®, is a herbal composite supplement that may provide supportive benefits to standard therapy for OA. It contains a patented curcuminoid preparation and extracts of the herbs *Harpagophytum procumbens*, *Boswellia serrata, Apium graveolens*, and *Zingiber officinale.* Curcumin is an active phenolic compound found in turmeric (*Curcuma longa*). In Tregocel^®^ curcumin is incorporated into a phospholipid complex (also trademarked as Meriva^®^), which was shown to be 29-fold more absorbed than the natural, unformulated curcuminoids [8]. It also contains an extract of *Harpagophytum procumbens*, with a few active ingredients including harpagoside as the primary one. Tregocel^®^ also contains an extract of *Boswellia serrata*, in which boswellic acids are primary active constituents. *Apium graveolens* (celery) seeds are another component, which contain volatile oils such limonene and selinene, flavonoid compounds, and celeroside glucosides and phthalide glycosides, as well as aromatic and lignin glucosides. Finally, Tregocel^®^ contains an extract of *Zingiber officinale* (ginger) with main phytochemicals such as phenols (gingerol, shogaols, and paradols), diarylheptanoids, gingerdiols, and sesquiterpenes [9].

Natural therapies involving supplementation with curcuminoids, boswellic acids, ginger, harpagosides, and luteolin (a type of flavonoid compound of celery) have been demonstrated to support pain relief and physical performance in OA. However, until now, these substances have not been evaluated clinically in a single dosage form or in the specific combination used in Tregocel^®^ [10].

This study aimed to provide clinical information on general and joint performance from individuals taking Tregocel^®^ as a dietary supplement in addition to standard therapies for symptomatic mild knee OA.

## 2. Materials and Methods

### 2.1. Study Design and Study Population

This was a multicenter, open-label, prospective, single-arm study which included a 1-week screening/run-in period, followed by 36 weeks of diet supplementation and a 4-week follow-up period after the last dose of supplementation. Five clinic visits were performed: baseline (visit 1), after 12 weeks (visit 5), after 24 weeks (visit 9), after 36 weeks (visit 13), and visit 14–4 weeks after the end of supplementation (additionally there were 9 remote visits). The study population consisted of participants with symptomatic mild knee OA requiring pharmacologic treatment for pain and meeting the following requirements at baseline: (1) presence of pain in the target knee at least half of the days in the past month (target knee was defined as the one with worse pain; if both knees presented the same level of pain, then the right knee was defined as the target knee); (2) maximal pain score ≥ 30 on a 100 mm VAS (Visual Analogue Scale) at screening and confirmed at baseline, and (3) use of pain killers as required (PRN) within the preceding month on at least 10 days (including at least 2 days in run-in period). After enrolment, both PRN treatment and regular OA treatment were able to be adjusted by an investigator to suit participant needs. Detailed inclusion and exclusion criteria are presented in Supplementary Table 1. During the study, patients were allowed to take acetaminophen (paracetamol) or NSAIDs. The following substances were prohibited: systemic steroids, intra-articular injections, glucosamine, chondroitin sulfate, diacerein, avocado/soya extracts, or other forms of herbal, vitamin, mineral, amino acid or related supplements.

### 2.2. Study Product

Subjects enrolled into the study received Tregocel^®^ supplementation in an open-label fashion. After enrolment at the baseline visit, 2 tablets of Tregocel^®^ were taken once daily with or after meals, for a duration of 36 weeks. The daily dose was equivalent to 1 g of curcuminoid-phospholipid complex, which is the primary active ingredient. Each Tregocel® film coated tablet contains curcuma phospholipid (500 mg; equiv. curcumin 90 mg), *Boswellia serrata* (Indian frankincense) gum oleoresin extract (500 mg; equiv. boswellic acids–81.25 g), *Harpagophytum procumbens* (devil's claw) tuber (500 mg), *Apium graveolens* (celery) seed (500 mg), and *Zingiber officinale* (ginger) rhizome (165 mg).

### 2.3. Study Objectives

The primary study objective was to assess physical performance with 6-minute walk test (6MWT) in participants with symptomatic mild knee OA taking standard therapy with Tregocel^®^ supplementation. The secondary objectives were to assess perception of pain, general performance, the need for standard pharmacological treatment, and the safety profile of Tregocel®.

### 2.4. Study Assessments and Procedures

Subjects with OA involving other joints were allowed, and providing pain in the target knee was the most predominant OA symptom, with no expected impact of pain in other OA locations on the primary outcome measure. Data from the subject medical history, including ongoing treatment and/or therapies and medications, were gathered. Osteoarthritis severity was assessed based on Kellgren–Lawrence classification (grades 0–4). Pain perception was assessed using a VAS scale, where 0 represented “no pain,” and 100 represented “extreme pain.” Subjects rated the perception of maximal pain during the last 24 hours on a daily basis and at clinic visits. For physical performance, 6-minute walk test (6MWT) and heel-thigh distance flexion test were performed during clinic visits, based on recording of the distance covered (in meters) during 6 minutes of walking in a straight line. In heel-thigh distance flexion tests, the investigator evaluated the angle and distance between heel and thigh at maximum knee flexion, in both supine and prone positions. For assessment of physical and general performance, the Western Ontario and McMaster Universities Osteoarthritis Index (WOMAC) questionnaire was also used. WOMAC is a widely used, proprietary health status questionnaire used to evaluate the condition of patients with OA of the knee and hip, including pain (5 items), stiffness (2 items), and physical functioning (17 items). The translated and validated Polish version of the WOMAC (VAS-based) was used in this study, with permission from Prof Nicholas Bellamy, University of Queensland, Herston, Australia (copyright holder, http://www.womac.org).

Vital signs were measured after 5 minutes of rest and included temperature, systolic and diastolic blood pressure, and heart rate. Additionally, standard clinical laboratory tests were performed as follows: haematology (red blood cell count [RBC], white blood cell count [WBC], haemoglobin, haematocrit, and platelets count), chemistry (aspartate aminotransferase [AST], alanine aminotransferase [ALT], total bilirubin, phosphates, creatinine, sodium, and potassium), C-reactive protein as an inflammation parameter, and urinalysis.

### 2.5. Statistics

A sample size of 150 was calculated as sufficient to determine a statistically significant mean difference of approximately 30% of standard deviation, assuming use of paired Student's *t*-test, at a significance level *α* = 0.05 and power of 90%. Continuous data are presented as mean and standard deviation (SD) for normally distributed variables and median with 1^st^ and 3^rd^ quartiles in case of nonnormally distributed variables. Categorical data are presented as numbers and percentages. Distribution of categorical variables was compared between time points using McNemar's test. The distribution of continuous variables was first evaluated with the Shapiro-Wilk test, then the normally distributed variables were compared between two time points using the paired Student's *t*-test; otherwise, the Wilcoxon test for paired data was used. To assess time effect on efficacy measure values in repeated measures design in case of analysing more than two time points, Skillings-Mack test was applied. The significance level was set at *α* = 0.05. Two-sided tests were used. Statistical analyses were performed with the statistical package R, version 3.4.1 (R Foundation for Statistical Computing, Vienna, Austria).

### 2.6. Ethical Consideration

The study conformed to the STROBE statement for reporting of observational studies. Local Ethics Committee in Wroclaw (Poland) approved the study (No 08/03/2018). The study was conducted in accordance with the Declaration of Helsinki. It was registered at ClinicalTrials.gov (NCT03636035).

## 3. Results

### 3.1. Study Population

**Figure 1:**
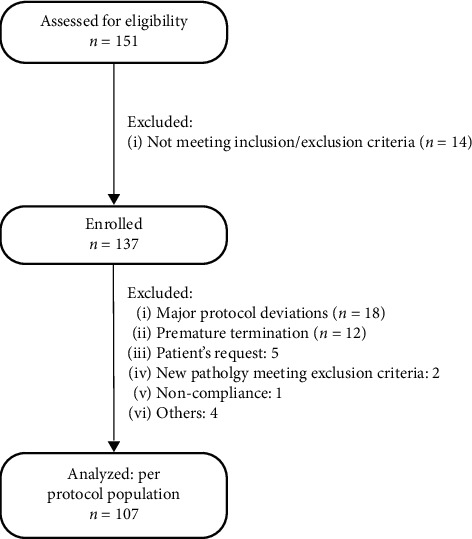
Study flowchart.

Between January and April 2019, a total number of 151 participants were screened across 8 different investigation sites across Poland. Of those screened, 137 were enrolled, with 107 participants completing the 36-week Tregocel^®^ intervention. This group was subsequently analysed as the per protocol population. The study flowchart is presented in Figure 1. Mean observation time was 291.1 ± 7.7 days. The mean age was 59.7 ± 10.8 years, and there were 68.2% (*n* = 73) of women in the study population, with right knee in 62.6% (*n* = 67) identified as the target knee. The median duration of OA was 1.8 [1.2, 4.6] years (min 0.4, max 17.7 years). The distribution of knee OA severity as per Kellgren–Lawrence classification was found to be as follows: class 0–4.7%, class 1–50.5%, and class 2–44.9%. Detailed dataset characteristics are presented in Table 1. Of the compliant individuals (98.1% of the per protocol group), compliance was equal to or greater than 94.5%.

### 3.2. Physical and Global Performance

**Figure 2:**
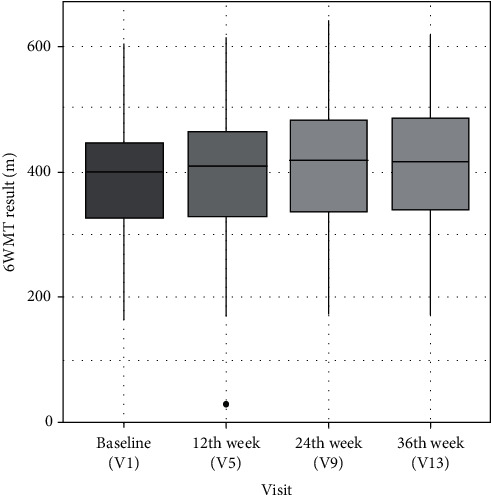
Results of 6-minute walk test (6MWT) at visit 1, visit 5 (after 12 weeks), visit 9 (after 24 weeks), and visit 13 (after 36 weeks).

The primary endpoint was to assess physical performance with 6MWT in subjects with symptomatic mild knee OA taking standard therapy with Tregocel^®^ supplementation. At baseline, the mean 6MWT result was 382.8 ± 88.1 m, and at the end of the study was 408.8 ± 96.3 m. The difference of 26.0 ± 30.4 m was statistically significant (*p* < 0.001, Student's *t*-test) (Figure 2).

**Figure 3:**
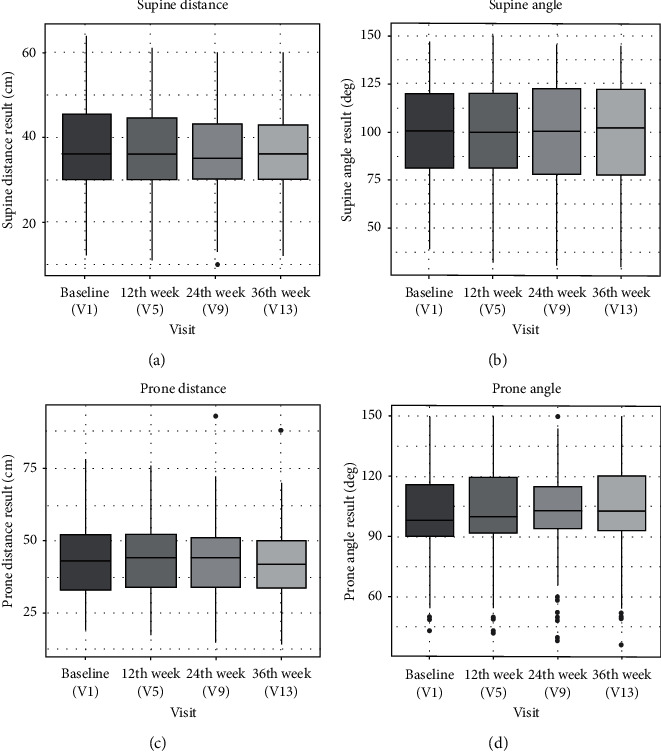
Results of heel-thigh distance flexion test. Comparison of supine distance, supine angle, prone distance, and prone angle at visit 1, visit 5 (after 12 weeks), visit 9 (after 24 weeks), and visit 13 (after 36 weeks).

Regarding heel-thigh distance flexion test, distances (supine and prone) were significantly improved from visit V5 comparing to baseline V1, with further differences observed also at visits V9 and V13 (*p* < 0.001, Skillings-Mack test) (Figure 3). Supine and distance angle values did not significantly change in time.

**Figure 4:**
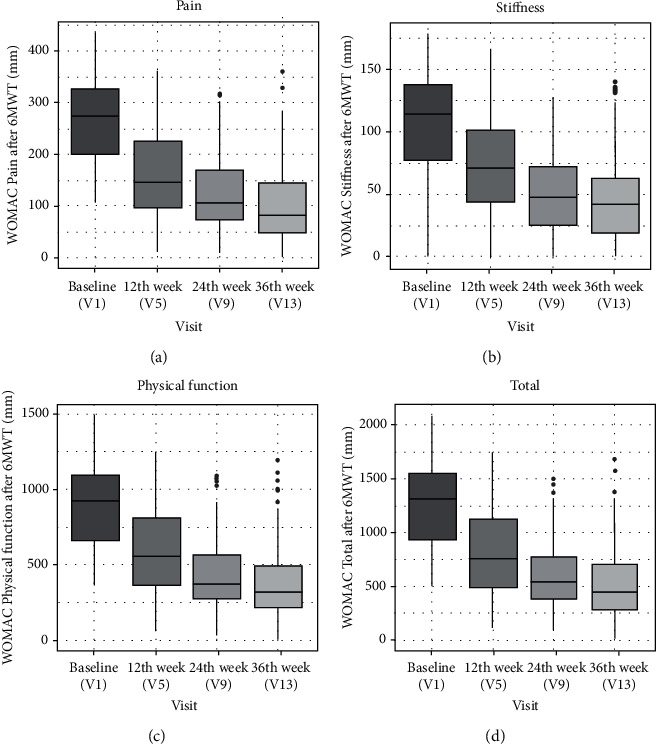
Results of WOMAC questionnaire. Comparison of pain domain, stiffness domain, physical function domain, and total at visit 1, visit 5 (after 12 weeks), visit 9 (after 24 weeks), and visit 13 (after 36 weeks). Results are presented as median with interquartile range (IQR).

WOMAC scores assessed before the 6MWT in all domains (pain, stiffness, physical function, and total) had improved progressively throughout the study. The scores on the subsequent visits statistically significantly decreased in time (*p* < 0.001 for all WOMAC scores, Skillings-Mack test). The level of decrease was the greatest on V5 (about 50% in all domains) and continued to improve in subsequent visits (Figure 4).

### 3.3. Pain Assessment and Concomitant Medications

**Figure 5:**
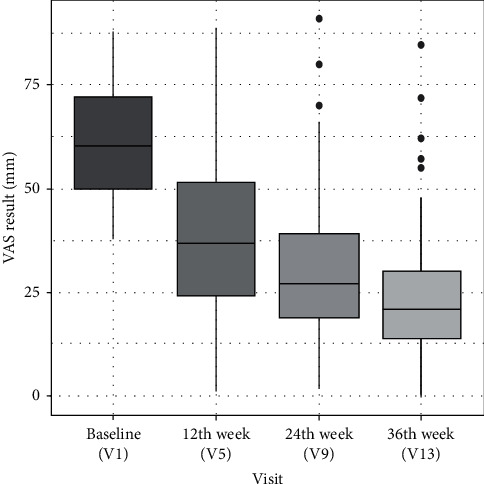
Pain assessment in VAS scale at visit 1, visit 5 (after 12 weeks), visit 9 (after 24 weeks), and visit 13 (after 36 weeks). Results are presented as median with interquartile range (IQR).

With the duration of the study, subjective pain levels significantly decreased from baseline [60.0 (IQR 50.0–72.0) at V1] to 37.0 (IQR 24.5–51.5) at V5, 27.0 (IQR 19.0–39.0) at V9 and 21.0 (IQR 14.0–30.0) at V13 (*p* < 0.001, Skillings-Mack test). The overall decrease was statistically significant and the value at the end of the study stood at approximately 30% of baseline pain scores (Figure 5).

As a consequence, the decrease in the administration of regular knee OA medications was also observed. At baseline, 99.1% of patients regularly took anti-inflammatory/analgesic drugs, whereas this progressively declined after 12 weeks (V5, 76.6%), 24 weeks (V9, 69.2%), and 36 weeks (V13, 55.1%).

### 3.4. Safety Conclusions

All patients who received at least one dose of Tregocel® were included in the safety analysis; therefore, the safety population counted 137 participants.

A total number of 32 AEs (adverse events) were reported in 25 (23.4%) subjects, including one (0.7%) SAE (serious adverse event). The SAE was associated with a right knee injury, and not related to the study product. In 25% of AEs, concomitant therapy was initiated; in 15.6%, supplement suspension/discontinuation was ordered; in 21.9%, other actions were taken; and in 37.5%, no actions were required to be taken. No vital signs or lab test abnormalities were found except for mild and transient increase in AST/ALT. A listing of all AEs is provided in Table 2.

## 4. Discussion

This was the first study to evaluate the safety and efficacy of the polyherbal product, Tregocel^®^, given as a dietary supplement in parallel with standard medications for mild knee osteoarthritis. Subjects in the per protocol group displayed improved functional capacity, confirmed in the distance in 6MWT and in the heel-thigh distance flexion test, decreased level of pain (VAS scale), and improvements in all WOMAC domains. These differences were observed as early as after 12 weeks of supplementation and improved even further with maximal effect at 36 weeks. Moreover, Tregocel® supplementation was characterized by a favourable safety profile.

With population aging, the problem of osteoarthritis will continue to grow, with the knee being one of the most common joints prone to injury. And this might predispose to osteoarthritis even more. Suter et al. [11] projected the cumulative incidence of symptomatic knee OA requiring total knee repair in the following scenarios: no prevalent or incident injury; isolated anterior acute ligament tear, surgically treated; isolated anterior acute ligament tear, nonoperatively treated; or a prevalent history or surgically treated anterior acute ligament and meniscal tear. The authors observed that the estimated lifetime risk of symptomatic knee OA was 34% for patients with an anterior acute ligament injury and meniscal tear, compared to 14% for the no-injury cohort. Anterior acute ligament injury without meniscal tear was associated with a lifetime risk of knee OA between 16% and 17%. Moreover, subjects in the anterior acute ligament injury and meniscal tear cohort developed OA approximately 1.5 years earlier (55.7 vs. 57.1) than patients without knee injuries. These results prompted us to investigate dietary supplements aimed to preserve knee joint health and function.

The results of this study with Tregocel® (improvement in walking distance, pain reduction, and improved general performance) were consistent with the observations of previous clinical studies involving the curcuminoids-phytosome complex used in the formulation [8, 12]. Curcuminoids are of special interest mostly due to unique properties of curcumin, which has been shown to downregulate the expression of matrix metalloproteinase-3 in OA. They also display antioxidant activity, which is associated with amelioration of joint tissue damage. As a result, curcumin has the potential to restrain inflammation and tissue damage in OA [13, 14].

Recently, Shep et al. [15] compared curcumin with diclofenac in patients with knee OA. One hundred and thirty-nine patients with knee OA were randomized to receive a curcumin 500-mg capsule three times daily or a diclofenac 50-mg tablet two times daily for one month. At days 14 and 28, patients receiving curcumin showed comparable betterment in pain severity and in Knee Injury and Osteoarthritis Outcome Score (KOOS) values, when compared with diclofenac. However, at day 7, patients in the curcumin group experienced a significantly larger increase in the number of episodes of flatulence compared with diclofenac (*p* < 0.01). At the end of the study, a weight-lowering effect (*p* < 0.01) and antiulcer effect (*p* < 0.01) of curcumin were registered. In the curcumin group, no patients required H_2_ blockers, and 19 patients in the diclofenac group (0% vs. 28%, respectively; *p* < 0.01). Moreover, adverse effects overall were significantly less frequent in the curcumin group (13% vs. 38%; *p* < 0.01). Other studies also confirmed the beneficial use of curcuminoids in knee OA management [16, 17].

In a systematic review, Onakpoya et al. [18] investigated the efficacy of curcuminoids administered orally in OA. The authors included seven studies with a total number of 797 patients with primarily knee OA. Compared with placebo, the use of curcuminoids significantly decreased knee pain (*p*=0.001) and improved quality of life (*p* < 0.001). Curcuminoid use was also associated with significant improvements in WOMAC total scores as well as with significant reductions in the use of rescue medication. No SAEs were reported.

Haroyan et al. [19] proved that curcuminoid complex extract from turmeric rhizome with turmeric volatile oil, combined with an extract of boswellic acids, performed better than placebo in physical performance tests and the WOMAC joint pain index, while when only the curcuminoid complex extract was used, it was more effective than placebo only in terms of physical performance tests. A further meta-analysis by Bannuru et al. [20] confirmed that curcuminoids and *Boswellia* formulations might pose a valuable additive to the knee OA treatment regimens. They effectively relieve pain symptoms and simultaneously reduce safety risks.

Recently, there was published an interesting study aimed to understand whether any pharmacokinetic interactions are among the major constituents of *Boswellia serrata* extract, curcumin, pine bark extract, and methylsulfonylmethane so as to provide information when considering the combination use of these supplements. The pharmacokinetics of each constituent was characterized, and there were no significant differences in the pharmacokinetic profiles of the constituents when administered as a combination, relative to the constituents when administered alone [21].

Safety analysis of Tregocel® supplementation was also favourable. There were 32 registered AEs (affected 23.4% of population) and they were reported, including one SAE (affected 0.7% of population). No deaths were reported. Only seven of the AEs were determined by the investigators to be related to the study product. Also, the increase in AST/ALT levels was transient and in the opinion of investigators was also not related to the study product.

Although this was the first clinical study to evaluate Tregocel® supplementation in OA, there are two main limitations. The first is the lack of a control group for comparison, and the second is the potential influence of concomitant drugs (e.g., NSAID) on the observed effects. While the use of standard medications had decreased during the course of the study, future randomized, placebo-controlled trials combined with regression analyses would be required to confirm the observed effects of Tregocel^®^ on OA symptoms.

This was the first study with the use of dietary supplement Tregocel® in subjects with mild knee osteoarthritis. During Tregocel® supplementation (36 weeks), subjects observed improved functional capacity confirmed in the distance in 6MWT and in the heel-thigh distance flexion test, decreased level of pain (VAS scale), and improved general performance (WOMAC score). These differences were observed as early as after 12 weeks of supplementation and improved even further with maximal effect at 36 weeks. Tregocel® supplementation also characterized a favourable safety profile. The results are encouraging and warrant verification in a randomized placebo-controlled study.

## Figures and Tables

**Table 1:** Baseline characteristics.

Parameter	Value
Demographics	
Mean age, mean (SD)	59.7 ± 10.8
Female, *n* (%)	73 (68.2)
Race – Caucasians, *n* (%)	107 (100)

Vital signs	
Weight (kg), mean (SD)	74.97 ± 11.97
Height (m), mean (SD)	1.66 ± 0.1
BMI (kg/m^2^), mean (SD)	27.12 ± 3.51
Sitting pulse (beats/min), mean (SD)	70.6 ± 6.8
Systolic blood pressure (mm Hg), mean (SD)	129 ± 9
Diastolic blood pressure (mm Hg), mean (SD)	78.7 ± 7.5

Knee osteoarthritis	
Duration (years), median (IQR)	1.81 (1.16, 4.58)
Mean observation time (days), mean (SD)	291.1 ± 7.7

Target knee	
Right, *n* (%)	67 (62.6)
Left, *n* (%)	40 (37.4)

Kellgren–Lawrence classification: *n* (%)	
0	5 (4.7)
1	54 (50.5)
2	48 (44.9)
3	0
4	0

Treatment: *n* (%)	
Anti-inflammatory/analgesic drugs	106 (99.1)
Physiotherapy	27 (25.2)
Orthopaedic supply	1 (0.7)

**Table 2:** Adverse events in the safety population (*n* = 137).

Adverse event	*N* (%) *N* = 32
Anemia	6 (4.4)
Diarrhea/vomiting	4 (2.9)
AST/ALT increase	4 (2.9)
Increase in C-reactive protein	3 (2.2)
Muscle stiffness/numbness	3 (2.2)
Stomach pain	3 (2.2)
Knee injury	2*∗* (1.6)
Upper respiratory tract infection	2 (1.6)
Bloating	1 (0.7)
Fracture of the right upper limb	1 (0.7)
Leg swelling	1 (0.7)
Leucopenia	1 (0.7)
Sciatica	1 (0.7)

*∗*Including one serious adverse event. ALT: alanine transaminase; AST: aspartate transaminase;

## Data Availability

The datasets used and/or analysed during the current study are available from the corresponding author on reasonable request.

## Supplementary Materials

Supplementary Materials The Supplementary Table 1 contains inclusion and exclusion criteria.
