# Supplementary material for: Prospective, Multicenter Evaluation of a Polyherbal Supplement alongside Standard-of-Care Treatment for Mild Knee Osteoarthritis
Source: Adv Orthop. 2021 May 7;2021:5589597. doi: 10.1155/2021/5589597 (PMC8124005; doi:10.1155/2021/5589597)
Supplement: Supplementary Materials — The Supplementary Table 1 contains inclusion and exclusion criteria. [file 5589597.f1.docx]

**Supplementary Table 1.** Inclusion and exclusion criteria

| **Inclusion criteria** | **Exclusion criteria** |
| --- | --- |
| At screening:   1. Able to understand study information and capable of giving signed informed consent which includes compliance with the requirements and restrictions listed in the informed consent form (ICF) and in this protocol. 2. Willing and able to comply with all study requirements, procedures and instructions of the site study personnel. 3. Male or female individual aged 40 years or older. 4. A female individual is eligible to participate if she is not pregnant, not breastfeeding, and declares intention not to become pregnant during the study duration if applicable (standard contraception is recommended for women with childbearing potential). 5. Individual with documented (in medical history with x-ray examination of target knee not older than 12 months) diagnosis of mild knee (including patello-femoral joint) OA (Kellgren-Lawrence classification ≤2) and clinical symptoms lasting more than 6 months prior to the Screening. 6. Individual with knee OA pain requiring pharmacologic treatment and meeting the following requirements:  - presence of pain in the target knee at least half of the days in the past month - maximal pain score ≥30 on a 100 mm VAS at Screening and confirmed at Baseline - PRN use of pain killers within last month on at least 10 days (at least 2 days in Run-in period) - stable OA treatment (oral and/or topical NSAIDs) as a regular pharmacologic treatment within 2 months prior to Screening.  1. Ambulatory with Eastern Cooperative Oncology Group (ECOG) performance status <2.   At baseline:   1. Participant providing completed Participant Diary for the Run-in period 2. Participant with at least 2 maximal pain VAS scores ≥30 on a 100 mm VAS during the 7-day Run-in period. 3. Stable regular OA therapy between the Screening visit and the Baseline visit. 4. PRN use of pain killers on at least 2 days in Run-in period. 5. Participant demonstrating understanding and compliance with study procedures in Screening and Run-in period, as per Investigator judgment (i.e. adequate completion of Participant Diary). | Medical Conditions:  1. Participants with secondary OA of the target knee.  2. Clinically apparent tense effusion of the target knee.  3. Significant valgus/varus knee/foot deformities, ligamentous laxity, or meniscal instability as assessed by Investigator.  4. Changes in regular OA therapy during Screening (to be verified at the Baseline visit)  5. Chronic diseases (i.e. systemic inflammatory diseases) which may require treatment with systemic steroids.  6. Progressive serious medical conditions (such as cancer, AIDS, or end-stage renal disease).  7. Severe organ dysfunction (liver function tests (aspartate transaminase [ASPAT] and alanine transaminase [ALAT]): >2.5 upper limit of normal [ULN], renal: creatinine > 2 ULN).  8. Cardiac insufficiency (New York Heart Association [NYHA] >2).  9. History of gastrointestinal ulcer or bleeding.  10. Any significant medical conditions (e.g. significant psychiatric or neurological disorders, active alcohol / drug abuse, diabetes, cardiovascular diseases, severe hypertension, blood and coagulation disorders, liver and gallbladder diseases, etc.) or other factors (e.g., planned relocation) which in the judgment of Investigator may interfere with the study procedures, safety, compliance or overall participation in the study.  11. History of allergic reactions or intolerances to any of Tregocel® ingredients.  Prior/Ongoing Therapy:  12. Any intra-articular injections in the target knee within 9 months prior to Screening.  13. Arthroplasty of the target knee at any time or any other previous surgery in the target knee within the 6 months prior to Screening, or planned surgery throughout the duration of the study.  14. Use of new OA treatment within 2 months prior to Screening including supplementation for OA (i.e. glucosamine, chondroitin sulfate, diacerein, avocado/soya extracts or other therapies); Participant using other OA dietary supplement(s) and participants using drugs which could have possible interactions with Tregocel® ingredients like anticoagulants (e.g. warfarin); anti-hypertensives; beta-blockers (e.g. talinolol); anti-diabetic drugs; sulfasalazine; P-glycoprotein substrates are excluded from the trial participation.  Prior/Concurrent Clinical Study Experience:  15. Participants should not participate in other clinical studies after signing informed consent.  Diagnostic assessments:  16. Arthroscopy within 9 months prior to Screening.  Other Exclusions:  17. Participants with body mass index (BMI) <18.5 kg/m^2^ and >35.0 kg/m^2^. |
